# Epigenetic and Metabolic Regulation of Macrophages during Gout

**DOI:** 10.3390/gucdd1030013

**Published:** 2023-07-12

**Authors:** Isidoro Cobo, Jessica Murillo-Saich, Mohnish Alishala, Monica Guma

**Affiliations:** 1 Department of Cell and Molecular Medicine, University of California San Diego, La Jolla, CA 92093, USA; 2 Department of Medicine, University of California San Diego, La Jolla, CA 92093, USA; 3 VA Healthcare Service, San Diego, CA 92161, USA

**Keywords:** metabolite, lipids, epigenetic, histone, transcription factor

## Abstract

The analysis of metabolite mediators has allowed a broader understanding of disease mechanisms. Experimental evidence indicates that metabolic rewiring is a key feature of inflammatory cells to restore tissue homeostasis upon damage. Over the last two decades, next-generation sequencing techniques have offered the possibility of looking at the genome-wide effect of the exposure of inflammatory cells to external stimuli. During gout flares, monosodium urate crystals activate a distinct metabolic profile and inflammatory transcriptional program in inflammatory cells. The extracellular signals are transduced through distinct signalling pathways, which are regulated by non-coding RNA and DNA sequences, and modification of histones. During response to inflammatory stimuli, changes in the abundance of metabolic mediators can regulate the activation of histones and of chromatin remodellers. The interplay between metabolic changes by MSUc, the regulation of epigenetic changes and the activation of transcription factor networks in inflammatory cells remains unknown. A better understanding of the interplay between metabolites and how it alters inflammatory response may provide novel insights into disease mechanisms during gout. In this review, we aim to provide a deeper understanding of the current view of how metabolic deregulation could alter the epigenetic landscape of inflammatory cells during gout.

## 1. Introduction

Over the last five decades, the working hypothesis has been that most illnesses have a genetic disorder in their origin [1–3]. Vigorous efforts in large-scale, population-wise studies have aimed to find gene susceptibility to disease by genome-wide association studies (GWAS). Even though GWAS have generated essential insights to understand the underlying mechanisms of some diseases [4,5], they have resulted in only a few susceptibility genes being identified [6,7]. This indicates that additional factors such as chronic environmental causes [8–10], an altered epigenome, and the contribution of metabolites and microbiome could take part in the onset and progression of human diseases [8–10].

Gout is the most common inflammatory arthritis worldwide, and its incidence is rising in developed and underdeveloped countries [11]. Gout is caused by the deposition of monosodium urate crystals in the joints in patients with persistent hyperuricaemia (HU) [12–16]. Besides the local clinical manifestations in the joints, gout is associated with many other systemic complications, from renal disease [17,18] to cardiovascular disease [19–22], diabetes [23], and metabolic syndrome [24,25]. Since the deregulation of urate metabolism is at the heart of gout, it is vital to understand genetic conditions [26,27] and environmental or behavioural exposures such as diet [28–33] that modify blood urate levels and underlying molecular mechanisms [28,29,31].

Together with the acute inflammatory reaction, gout attacks are accompanied by an altered local and systemic metabolomic profile [34–36]. Diverse significantly dysregulated pathways have been described in individuals with hyperuricaemia and patients with gout compared to normouricaemic controls, among which arginine metabolism [37] and other amino acids [38] appeared to play a critical role. Lipid and carbohydrate metabolism are other proposed dysregulated pathways [39,40]. Finally, a significant increase in leukotriene B4 (LTB4) in plasma associated to an increased transcriptional level of 5-lipoxygenase in whole blood cells was described in patients with acute gout flares [41].

Metabolites are the substrate, intermediate, or final products of metabolic reactions that drive the function of a given cell in a particular time and context. Therefore, metabolites provide essential information about the connection between gene expression and the environment, and, as such, they are elegant disease biomarkers [42]. Over the last decade, metabolomics has extensively characterized metabolites and metabolic pathways in many biological systems, providing novel opportunities to understand disease mechanisms and untangle the cause of complex diseases such as cancer [43,44], diabetes [45], cardiovascular [46–48], and other types of inflammatory arthritis [49–51]. In addition, it has been suggested that the metabolic deregulation observed during gout could contribute to kidney and cardiovascular disease [24,52,53]. Metabolomics offers, combined with other omics (genomics, transcriptomics, proteomics), an increasing number of biomedical applications, from disease diagnosis to patient monitoring, personalized drug treatments, and predicting drug response [54]. Recently, the application of machine learning to perform automated identification and quantification of novel metabolites has provided a road map of metabolic deregulation in various biological scenarios [55,56]. In this Review, we will provide a general overview of how metabolic changes affects histone lactylation and acetylation macrophages during response to external stimuli. We also explain how metabolic changes affect AP-1 transcription factor binding in macrophages by MSUc. Finally, we will present a hypothetical novel mechanism of inflammation resolution by changes in lipid metabolism during gouty inflammation by MSUc.

## 2. From Metabolomics to Epigenetics and Transcription Factor Binding: Coupling Environmental Changes to Molecular Phenotypes

The biochemical actions of metabolites go far beyond their role in biosynthesis and bioenergetic processes. In the last decade, metabolomics has shed light on how metabolites alter gene expression and contribute to dictating biological phenotypes [54]. The well-characterized role of metabolites in regulating the epigenetic landscape of embryonic stem (ES) cells [57–60] was followed by an increasing interest in how metabolites alter the immune cell phenotype, also named immunometabolism. The field of immunometabolism has emerged as a critical tool for understanding how metabolic changes can modulate immune cell response [61–63]. Moreover, we propose that the connection between epigenetics and metabolism will provide new avenues to understand disease predisposition and to develop personalised treatment [64,65]. The assembly of eukaryotic genomes is accomplished by a complex of octamers of histones that bind to the DNA. The segment of DNA wrapped around the histone octamer in the nucleosomes presents a barrier for the binding of transcription factors (TFs), transcription initiation complexes, and other transcriptional regulators. Therefore, the interaction of TFs with histone modifications plays a crucial role in integrating a finely tuned gene expression program [66–68]. Some post-translational modifications of histones and epigenetic regulators impact the inflammatory reaction during response to external stimuli [69,70]. For instance, whereas genome-wide remodelling of acetylation of lysine 27 of H3 (H3K27ac) is seen specifically in distal regions, changes in methylation of lysine 4 of H3 (H3K4me3) are mainly detected in genomic regions close to the transcription start site and regulate macrophages’ response to microenvironmental signals [69–73]. During the last decades, the development of next-generation sequencing (NGS) techniques has allowed profiling the histone landscape and transcription factor binding genome-wide to understand the dynamic mechanism of gene expression during homeostasis and disease [74,75]. In addition, NGS techniques have allowed an advance which links genetics with environment and disease, revolutionizing the way we carry out science [76,77].

Macrophages represent an elegant model for understanding histone dynamics, transcription factor recruitment, and changes in gene expression during signal transduction by environmental signals [69,78,79]. These environmental signals are integrated with intracellular signalling pathways that regulate non-coding DNA regulatory elements (RE), termed enhancers, and promoters, to control macrophage phenotypes. The accessibility of DNA binding sites in the regulatory elements controls the ability of TFs to produce a spatiotemporal-specific and context-specific transcriptional output. Macrophage phenotypes are regulated by activation of distinct inflammatory programs determined by the response of various TF to external signals from the cellular context [69,78,79].

### 2.1. Histone Lactylation Contributes to Establishing an Inflammation Resolving Program in Macrophages

In the immunometabolism field, there has been emerging interest in understanding the molecular consequences of metabolic imbalance in regulating histone activity through changes in post-translational modifications (PTMs) [80]. Histone lactylation is one of the most compelling cases of epigenetic modification by changes in metabolic balance in macrophages. The Warburg effect was first described in cancer cells, where glycolysis is highly upregulated; therefore, cancer cells produce large amounts of lactate [81–83]. Initially, lactate was considered a by-product of the glycolytic activity of the cell. However, more evidence suggests that lactate is involved in various cellular processes in health and disease [84–86], including activating the Krebs cycle [87,88] and acting as an extracellular signalling molecule allowing the intercommunication of neighbouring cells [89]. In 2019, Zhang and colleagues demonstrated that lactate could modify histones in macrophages, a process named histone lactylation [90]. During the response to a pro-inflammatory perturbation, macrophages produce larger amounts of lactate that, among other molecular consequences, leads to histone lactylation over a subset of inflammatory genes associated with establishing an anti-inflammatory gene expression program and genes related to facilitating the resolution of inflammation [90]. Zhang’s work has been followed up by other studies supporting the hypothesis that dynamic histone lactylation by lactate is a hallmark of metabolic rewiring and a crucial mechanism of gene expression in macrophages [91,92]. Interestingly, histone lactylation is not observed over the promoter regions of inflammatory genes and does not alter the expression of cytokines and other proinflammatory genes, indicating specificity for a subset of anti-inflammatory genes. The current view is that to react against an inflammatory insult the cells need to use glucose via anaerobic glycolysis to activate an inflammatory gene expression program at early time points. The sustained activation of glycolysis leads to increased intracellular lactate levels, which induces lactylation of the histones recruited over the promoter of anti-inflammatory/resolution genes. Histone lactylation is a hallmark of an anti-inflammatory phenotype in macrophages that establishes resolution of inflammation. Importantly, we and others have demonstrated that stimulation of macrophages with MSUc leads to increased glycolytic metabolism, including higher intracellular lactate levels [34–36]. Thus, histone lactylation to activate anti-inflammatory genes could be part of the underlying mechanism observed during gout flares that leads to resolution. In addition, we speculate that the imprinting of an anti-inflammatory epigenetic and transcriptomic signature during gout flare must occur to avoid the apoptosis of inflammatory cells and the destruction of the synovial membrane of the joint tissue MSUc and promote resolution of the flare. This mechanistic role for the so-called “reparative inflammation” has been extensively studied in highly proliferative tissues such as gut and liver epithelial cells [93], but common molecular behaviour highlights its importance in other tissues. As they are involved in any inflammatory disease, there has been great interest in understanding the role of innate immune cells, mainly macrophages, in regulating resolution of inflammation [94–97]. Therefore, we hypothesise that histone lactylation would have a crucial role in regulating macrophage response to MSUc. However, supporting experimental data are required to confirm this hypothesis.

### 2.2. Histone Acetylation Takes Charge of the Dynamic Enhancer Activation in Macrophages in Response to External Stimuli

Besides glycolysis, another important metabolic pathway is the Krebs cycle. The Krebs cycle is fuelled by acetyl-CoA produced from glucose or fatty acids and can lead to acetylation. Interestingly, MSUc leads to increased glycolysis, with accumulation of citrate and succinate [35], which together with itaconate can modify proteins in the context of immunity and inflammation [98,99]. For instance, itaconate was shown to modify cysteines on a range of target proteins, with the modification being linked to a functional change [98]. In addition, acetyl-CoA can produce acetylation and deacetylation of histones and it represents one of the most widely used histone modifications to understand the epigenetic activation of macrophages during inflammation [69,70,79]. Two main protein families, the histone acetyltransferases (HATs) and the histone deacetylases (HDACs), dynamically and reversibly control the acetylation state of histones and, in consequence, are important regulators of the context-specific responses of macrophages [100], and help to decipher molecular phenotypes of macrophages during disease. This dynamic epigenetic response has been largely studied in enhancer regions by using H3K27ac as a read-out of enhancer activity in the context of macrophage activation by TLR agonists and other ligands [70,78,101], but the enhancer landscape induced by MSUc in macrophages remains unknown. In vitro studies of mouse macrophages indicate that most enhancers are recognised by a combination of TFs that collaboratively interact with each other to bind to specific DNA sequence motifs in regulatory regions of the genome. Of these TFs, the current view is that the transcriptional output of a repertoire of enhancers in macrophages is dictated by the contribution of lineage-determining transcription factors (LDTFs), including PU.1 and the AP-1 family, and signal-dependent transcription factors (SDTFs), including nuclear receptors (NR) and NFKB and interferon regulatory factors (IRFs), among others [78,79,102]. Of note, interestingly, whereas the promoter of genes induced by LPS in macrophages is enriched in IRFs, NFKB and STAT motifs, the promoter of genes induced by MSUc is enriched in DNA motifs for AP-1, MITF/TFE, NR, and circadian clock regulators, indicating that a different combination of TFs takes charge of the epigenetic activation of macrophages by MSUc [35]. Moreover, the genes uniquely upregulated by MSUc belong to signalling pathways related to NRs signalling and transcription and activation of circadian clock regulators. However, this in silico prediction of putative binding of TFs to the DNA requires empirical validation to identify the motif enrichment in regions of open chromatin with increased enhancer activity. However, the changes in the epigenetic landscape of macrophages by MSUc and its contribution to altering inflammatory programs of gene expression remain to be elucidated.

### 2.3. Differential Recruitment of Transcription Factor Binding to Genomic Regulatory Regions Regulates the Response of Macrophages to MSUc: The Case of the AP-1 Family

The integration of histone landscape and TF binding by ChIP-Seq analysis with gene expression by RNA-Seq is fundamental to dissecting macrophage cell phenotypes during gout flares. Our work and that of others demonstrate that in macrophages signalling pathways regulated by inflammatory molecules such as MSUc during gout flares are coupled to a battery of TFs whose ability to induce specific gene expression programs is dictated by the accessibility (“openness”) of the chromatin and the presence of particular motifs in the macrophage DNA genome [35,69,103]. Our in silico analyses of DNA motifs define the putative transcription factor families to regulate the transcriptional output of macrophages and offer candidates to understand the interplay between MSUc and the control of macrophage function. In line with this, we found that the promoter region of genes induced by MSUc in unprimed bone marrow derived (BMDM) mouse and human monocyte-derived (MDM) macrophages are mainly enriched in motifs for activator protein 1 (AP-1). The AP-1 is a dimeric family that includes members of the JUN, FOS, activating transcription factor (ATF), and musculoaponeurotic fibrosarcoma (MAF) protein families [104,105]. The different dimer compositions of AP-1 complexes determine the biological output of the process controlled by AP-1 [106,107]. Historically described during oncogenic transformation [104,108,109], AP-1 members are abundant in macrophages, regulating molecular phenotypes and contributing to macrophage function. Increased JUN upregulation and phosphorylation by MSUc via JNK and increased JUN recruitment to the promoter of metabolic genes are required for the response of macrophages to MSUc. Molecular or pharmacological reduction in JNK-JUN activity modifies the epigenetic landscape; it ameliorates the induction of metabolic genes and metabolic changes, including a reduction in lactate, indicating that JUN’s role during gouty inflammation by MSUc goes beyond its role in activating an inflammatory gene expression program. The sustained activation of JUN and JNK and the altered metabolic program when JNK-JUN activity is compromised could indicate that JUN and its downstream target genes are involved in the later stages of the response of macrophages to MSUc by regulating metabolic mediators.

### 2.4. Epigenetic Changes by Higher Soluble Urate Levels in Myeloid Cells

Hyperuricaemia is the main risk factor for gout flares [12–16]. High levels of urate induce IL1b and IL6 production by monocytes and reduced levels of IL-1 receptor antagonist (IL-1Ra) [110]. Moreover, ChIP-Seq data on H3K27ac and H3K4me3 show that some inflammatory genes such as *Il1a*, *Il1b* displayed increased enrichment in H3K27ac and H3K4me3 by urate [110], which indicates that soluble urate can alter the epigenetic landscape of inflammatory genes in myeloid cells [111]. Together with changes in histone modifications, elevated serum urate alters the DNA methylation profile of circulating inflammatory cells including the glucose transporter *SLC2A9*, the amino acid transporter *SLC7A11* and the amino acid biosynthesis gene *PHGDH* [112]. Interestingly, SLC2A9 is a known urate transporter that regulates serum urate concentration and excretion during gout [113], indicating that epigenetic gene deregulation may provide information about genetic traits in hyperuricaemia and gout.

## 3. Lipidomics and Gout, Signalling Pathways in the Resolution of Inflammation by Macrophages

The deposition of MSUc in the joints causes a self-limited, acute inflammatory reaction. The effect MSUc during gouty inflammation offers a suitable system to understand anti-inflammatory programs of gene expression in macrophages. The original view was that biological systems resolve inflammation by diluting proinflammatory mediators that eventually restore tissue function. This view has been surpassed, thanks to the work of Dr Charles N. Serhan and others, by a more active notion where macrophages and other cell types produce specialized pro-resolving mediators (SPMs) and other anti-inflammatory oxylipins to counterbalance the initial wave of proinflammatory signals to prevent surplus inflammation and subsequent tissue damage [114–119]. Even though SPMs are oxylipins widely studied in the context of inflammation and have been primarily studied in other inflammatory diseases, including lung disease [118,120] and cancer [121], the role of SPMs in the resolution of gout flares remains unknown. SPMs, resolvins, protectins, and maresins are derived mostly from alpha linolenic acid (α-LA), which is an omega-3 essential fatty acid (EFA) from green leafy vegetables, flax and chia seeds, and walnuts. Omega-6 EFA are generally generated by linoleic acid (LA) from vegetable oils, meats, and eggs. Some omega-6 lipids, such as lipoxins, PGJ2, and PGB2, are also considered anti-inflammatory molecules [122].

The central catalytic enzymes involved in the generation of STMs are phospholipases (PLA)2 and lipoxygenases (LOX). The time course of biosynthesis and bioavailability of SPMs dictates their molecular function to ensure a cell type and context-specific response. In macrophages, lipoxin A4 (LXA_4_), protectin D1 (PD1) and resolvin D1 (RvD1) are involved in the clearance of apoptotic neutrophils and other polymorphonuclear cells [123–125]. Regardless of the subtype, SPMs exert their biological activity upon binding with high affinity to specific cognate receptors. Over the last years, the receptors for some of the SPMs have been characterized. LXA_4_ binds and signals through the LXA_4_ receptor (ALX or formyl peptide receptor(FPR2)) [126], RvE1 through chemokine-like receptor 1 (CMKLR1) [127], RvD1 through G protein-coupled receptor GPR32, and RvD2 through GPR18 [128,129]. Therefore, changes in EFA, an altered expression of the enzymatic cascade that bio-converts EFA to SPMs and other anti-inflammatory oxylipins, or changes in the expression and availability of any of the receptors will impact the activity of SPMs during the resolution of inflammation. Interestingly, the treatment of mice with MSUc results in an increased production of prostaglandins and other oxylipins suggesting that oxylipin metabolism could be also involved in limiting the duration of gouty inflammation by MSUc [130,131]. Below we will review some of the mechanisms that could contribute to regulate SPM production in macrophages during gout flares.

### 3.1. Phospholipases A2

Phospholipase A2 (PLA2) encompasses a superfamily of enzymes with more than 50 members, whose expression and activity dictate a cell-specific and temporal response [132–135]. PLA2 is the first enzymatic machinery in the metabolism of SPMs. Therefore, extensive work has been put into understanding PLA2 regulation during inflammatory processes in macrophages [136]. PLA2 enzymes can act as degradative, biosynthetic (when coupled to an acetyltransferase) or as a signalling enzyme. This versatility of action, the high degree of functional redundancy, and their dynamic expression have made the PLA2 family challenging to ascribe to specific regulatory signalling programs. It is widely accepted that many different mechanisms, including increased [Ca^2+^] [137], ceramide phosphate [138], phosphatidylinositol [139,140], bisphosphate [141], and phosphorylation [142] activate PLA2. In addition, the transcription of the endogenous secretory phospholipase A2 group IIA (sPLA2-IIA) gene is regulated by the direct binding of CCAAT/Enhancer Binding Protein (C/EBP), NFKB, and ETS proto-oncogene TF (ETS) transcription factors to the *PLA2* regulatory region [143,144]. Interestingly, whereas *Pla2g4a* and *Pla2g5* are upregulated, *Pla2g15* is downregulated, suggesting a role of Pla2 transcriptional regulation in macrophages during gout.

### 3.2. COX and ALOX5/ALOX5AP

The next step in the formation of oxylipins associated to the resolution of inflammation involves the conversion of docosahexaenoic acid (DHA), eicosapentaenoic acid (EPA), and some products derived from AA by cyclooxygenases (COX) and lipoxygenases. MSUc stimulates COX-2 expression in peripheral monocytes, which correlated with the synthesis of pro-inflammatory oxylipins such as prostaglandin E2 (PGE2) and thromboxane A2 (TXA2) [145]. Leukotriene B4 (LTB4) was also relevant in the MSUc-induced maturation of IL-1b [146]. Of interest, PGD2 and 15d-PGJ_2_ had an anti-inflammatory role in animal models of MSUc-induced inflammation [147,148]. Of the genes coding for lipoxygenases, *ALOX5*/*Alox5* and Alox5 activating protein *ALOX5AP/Alox5ap* are the two most expressed in unprimed MDM and BMDM. The significance of ALOX5/ALOX5AP during gout is supported by a study by Luo and colleagues where after performing metabolomics of PUFA of patients with acute gout plasma validated in two independent cohorts, they found a higher increase in leukotriene B4 (LTB4), accounting for altered activity of lipoxygenase 5 [41]. Notably, stimulation with MSUc leads to downregulation of *ALOX5* in unprimed *MDM* and *BMDM* and downregulation of *Alox5ap* in unprimed BMDM [35], which is in accordance with a negative feedback mechanism of metabolic networks to regulate active metabolic pathways [149,150]. Importantly, *ALOX5/ALOX5AP* are JUN target genes, and treatment with JNK inhibitor SP600125 ameliorates the downregulation by MSUc, providing further evidence that ALOX5 and ALOX5AP repression by JUN could contribute to the formation of oxylipins during gouty inflammation. Interestingly, the expression of the main LXA_4_ receptor, *FPR2*, is downregulated in unprimed MDM stimulated with MSUc. Of interest, besides the participation of 5-LOX in inflammation by promoting the biosynthesis of leukotrienes, this enzyme possesses other non-canonical functions as transcriptional regulator in monocytic cells including the interaction with β-catenin, p53, and chromatin [151,152]. These results provide substantial evidence to suggest a role of signalling by LOX products and their downstream signalling during the resolution of gout flares.

### 3.3. Activation of Enzymatic Pathways by Damaged Subcellular Organelles

During the early stages of the acute phase of gout flares, ingested MSUc induces the rupture of lysosome in leukocytes and the release of the lysosomal content into the surrounding medium, which is a hallmark of damage induced by MSUc [153–157]. Aberrant lysosomal compartment leads to increased intracellular and extracellular [Ca^2+^] [158,159], which can activate PLA2 to release free fatty acids that fuel the synthesis of new pro- and anti-inflammatory oxylipins.

## 4. Conclusions

In this review, we have provided extensive evidence demonstrating the importance of metabolomic analyses in gouty inflammation. Although well implemented in the study of other pathologies, the knowledge of the metabolic contribution during the acute phase of gout flares is relatively scarce. We have decided to focus the scope of this review on the some of the possible epigenetic mechanisms underlying the activation of innate immune cells, mainly macrophages, by MSUc during the acute phase of gouty inflammation. Undeniably, other cell types are involved in regulating the response to MSUc. However, the degree of plasticity of macrophage epigenetic phenotypes [69,79], their bona fide cell ability to be involved in the phagocytosis of MSUc, and their involvement in the resolution phase of inflammatory processes make macrophages an elegant target to dissect molecular mechanisms in pursuit of novel therapeutical regimens [153]. In addition, the extensive knowledge of the epigenetics and transcriptomics of macrophages after perturbation makes it easier to ascribe epigenetic programs of gene expression associated with the stimulation by MSUc.

**Figure 1.**
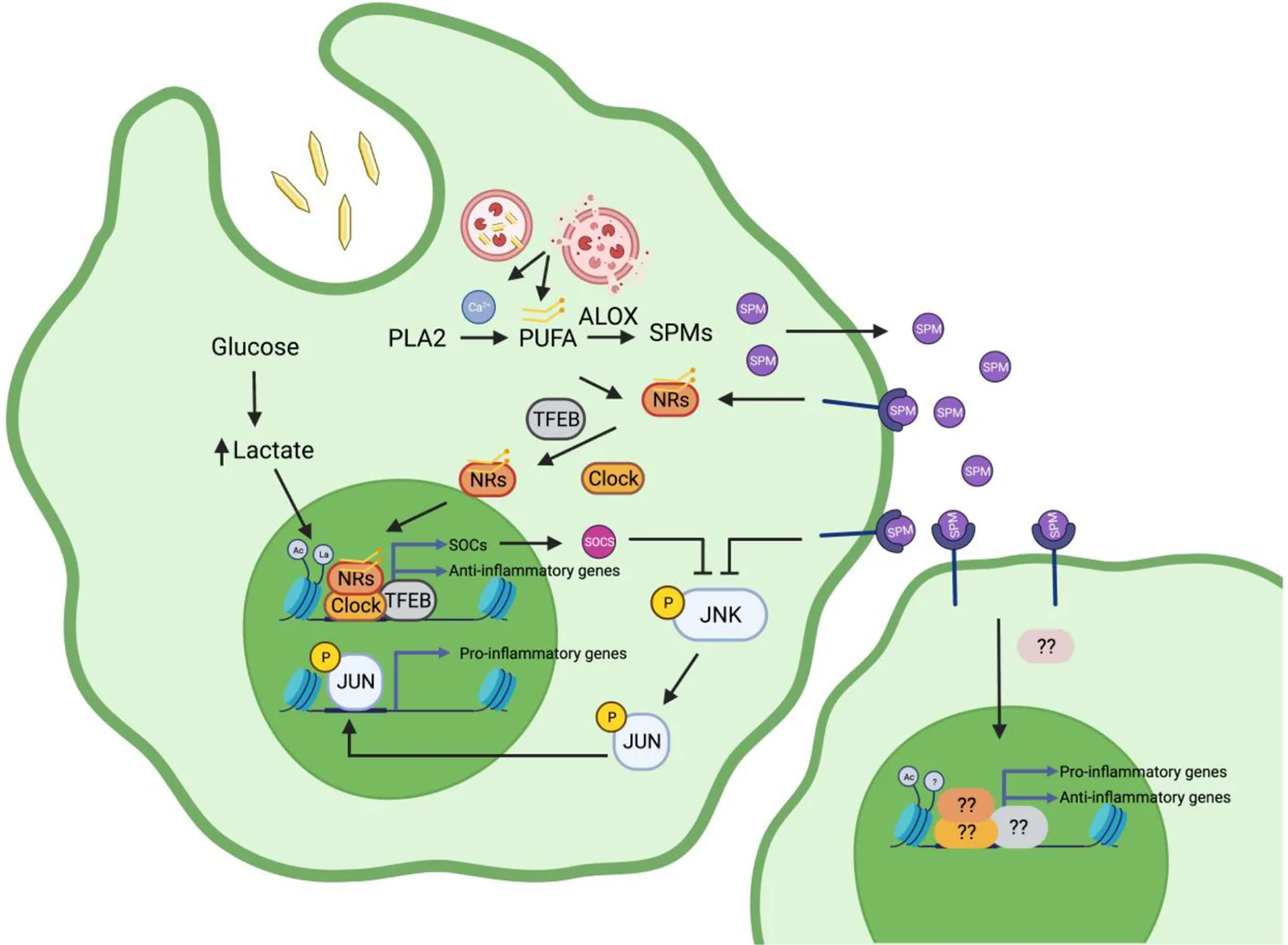
Mechanisms of macrophage activation during gouty inflammation. During gout flares, MSUc leads to a cell autonomous response in macrophages that is mediated by activation of transcription factors (TFs) including activation of nuclear receptors (NRs) upon binding to fatty acids, AP-1 via JNK signalling, MITF/TFEB, and circadian clock regulators (Clock). The recruitment of these TFs to genomic regions with altered histone landscape marked with histone lactylation (La), due to increased intracellular lactate, or H3K27ac (Ac) dictates the transcriptional response of macrophages by MSUc. On the other hand, local activation by MSUc leads to a non-cell autonomous activation of neighbouring macrophages mediated by a yet-unknown transcriptional and epigenetic mechanism. We hypothesise that the production of specialised pro-resolving mediators (SPM) by activated metabolism of polyunsaturated fatty acids (PUFA) via arachidonate lipoxygenases (ALOX) and intracellular calcium levels plays a crucial role in in activating a resolution program in macrophages during gout flares. Figure created with BioRender.com.

We have placed significant emphasis on the epigenetic activation of transcriptional programs by changes in metabolite composition as in the production of specialised molecules involved in the resolution of inflammation. Our view is summarised in Figure 1. In summary, during gout flares, macrophages respond to MSUc, activating first a cascade of pro-inflammatory signalling and then likely a more robust cascade engaged in the resolution of the inflammation. Whereas the “pro-inflammatory” phase enables macrophages to recruit other inflammatory cell types, the resolution phase might activate a vital signalling cascade to induce the phagocytosis of apoptotic cells and produce pro-resolution lipids to restore tissue homeostasis. In our view, the activation of a pro-inflammatory program by MSUc lies in the early activation of JNK-JUN and other AP-1 members [35]. The role of AP-1 in activating inflammatory programs is well known, and our data demonstrate that treatment with a JNKi ameliorates severe inflammation by MSUc in vitro and in vivo [35].

In parallel with the activation of inflammatory gene expression, we hypothesise that in macrophages during gouty inflammation, the resolution of inflammation is regulated at different levels. Increased lactate production through anaerobic glycolysis lactylates histone H3 and histone H4 could open the chromatin of the regulatory regions of anti-inflammatory genes and promote their transcription. The family of suppressors of cytokine signalling proteins (SOCS), including *Socs1*, *Socs3*, *Socs4*, *Socs5*, and *Socs7*, which are upregulated by MSUc [35], ameliorates the production of inflammatory cytokines and is a well-known negative regulator of JNK signalling [160–163]. The SOCS gene family is an example of many other anti-inflammatory genes induced by MSUc. Our data using a JNKi suggest that JNK and AP-1 also regulate the levels of some metabolites [35], including lactate, possibly through JUN binding to their promoter region, and could act as a TF effector downstream of histone lactylation. We acknowledge that the high levels of urate in patients with hyperuricaemia and gout can contribute to the deregulation of the epigenetic landscape of myeloid cells during gout flares. However, given the lack of strong deregulation of H3K4me3 or H3K27ac enrichment in monocytes exposed to urate [110], we hypothesise that the majority of epigenetic changes will be driven by the deposition of MSUc. On the contrary, we hypothesise that exposure to urate will impact the capacity of myeloid cells to respond to MSUc, priming myeloid cells to more exacerbated changes induced by MSUc. Moreover, it has been proposed that urate can induce immune memory in inflammatory cells [111], which is in accordance with our view of more dramatic epigenetic changes of macrophages by MSUc during gout. The proposed mechanisms of macrophage activation during gouty inflammation are summarised in Figure 1.

However, perhaps the most challenging thing will be to relate metabolites to their biological roles in regulating the response to MSUc. It is true that with machine learning techniques we have been able to narrow down the spectrum of action of a specific metabolite, but this is an ongoing area of research and needs to be improved. Integrating metabolomics with epigenomics, transcriptomics, and proteomics could help determine the relationship between gene expression, metabolite concentration, and biological function. Applying orthogonal approaches, including silencing gene expression by CRISPR-mediated knock-down, inhibiting enzymatic activity using chemical blockers or anti-metabolites, or targeting the immune response of macrophages, could help to provide novel mechanistic insights.
